# Simultaneous Inguinal Hernia Repair with Monofilament Polypropylene Mesh during Robot-Assisted Radical Prostatectomy: Results from a Single Institute Series

**DOI:** 10.3390/medicina59050820

**Published:** 2023-04-22

**Authors:** Giuseppe Chiacchio, Mattia Beltrami, Andrea Cicconofri, Carlotta Nedbal, Lucia Pitoni, Demetra Fuligni, Martina Maggi, Giulio Milanese, Andrea Benedetto Galosi, Daniele Castellani, Carlo Giulioni

**Affiliations:** 1 Urology Unit, Faculty of Medicine, School of Urology, Azienda Ospedaliera Universitaria Delle Marche, 60127 Ancona, Italy; 2 Department of Maternal Infant and Urologic Sciences, Sapienza University of Rome, Policlinico Umberto I Hospital, 00161 Rome, Italy

**Keywords:** prostate cancer, robot-assisted prostatectomy, robotic surgery, inguinal hernia, inguinal hernia repair, complications, uro-oncology

## Abstract

*Background and Objectives*: Inguinal hernia (IH) is a usual finding in men with prostate cancer (PCa) due to their similar risk factors, such as age, gender, and smoking. This study aims to present a single institution’s experience with simultaneous IH repair (IHR) and robotic-assisted radical prostatectomy (RARP). *Materials and Methods*: We retrospectively reviewed 452 patients who underwent RARP between January 2018 and December 2020. A total of 73 patients had a concomitant IHR with a monofilament polypropylene mesh. Patients with bowel in the hernia sac or recurrent hernia were excluded. *Results*: The median age and the American Society of Anesthesiologists (ASA) score were 67 years (inter-quartile range (IQR) 56–77) and 2 (IQR 1–3), respectively. The median prostate volume and preoperative prostate-specific antigen (PSA) were 38 mL (IQR 25.0–75.2) and 7.8 ng/mL (IQR 2.6–23.0), respectively. The surgery was successfully performed in all cases. The median overall and IHR operative time were 190.0 (IQR 140.0–230.0) and 32.5 (IQR 14.0–40.0) minutes, respectively. The median estimated blood loss and length of hospital stay were 100 mL (IQR 10–170) and 3 days (IQR 2–4), respectively. Only five (6.8%) minor complications occurred after surgery. At the 24-month follow-up, no cases of mesh infection, seroma formation, or groin pain were recorded. *Conclusions*: This study confirmed the safety and efficacy of performing simultaneous RARP and IHR.

## 1. Introduction

Prostate cancer (PCa) is the second most common cancer affecting men, with an estimated 1.4 million diagnoses worldwide in 2020 [1]. About one-third of patients with localized PCa receive surgery (i.e., radical prostatectomy (RP)) as primary treatment [2]. Although the European Urological Association (EAU) guidelines recommended informing PCa patients who are candidates for surgery that no surgical approach (open versus laparoscopic versus robot-assisted) has clearly shown superiority in terms of both functional and oncologic results, robot-assisted RP (RARP) has become the most frequently used technique for the surgical management of localized PCa [3,4,5]. Since its introduction in 2000, RARP has shown advantages primarily in the peri-operative setting (e.g., in terms of reduced blood loss, pain, length of hospital stay, and earlier recovery time), and now up to 90% of RPs are being performed robotically worldwide [6].

Inguinal hernia (IH) is a common condition with a prevalence of 25% in men, which accounts for most abdominal wall hernias (75%) and almost all groin hernias (97%) [7]. Inguinal hernia repair (IHR) is one of the most performed surgeries worldwide [8]. The main risk factors for hernia occurrence in males are aging and smoking, which are similar to those of PCa [9]. In addition, IH is a common finding in men diagnosed with PCa. In a study by Watson et al., an incidence rate of 13% was reported for asymptomatic IH detected during RP [10]; similarly, in the case series of Futuka et al., 20% of PCa patients undergoing RP were found to have sub-clinical IH [11].

Conversely, IH development has also been reported as both a short- and long-term complication following RP. Regan et al. showed that the incidence of IH after RP was 12%, significantly higher than the general male population of the same age [12]. The aetiology of post-RP IH is still poorly understood, and no intra-operative potential risk factors were found to be associated with its occurrence [13,14]. Therefore, treatment of asymptomatic IH diagnosed intra-operatively should be taken into consideration. A recent review confirmed the safety and feasibility of IHR during RP, although the use of a mesh may be associated with complications, such as infections, post-operative pain, and bowel adhesions [15].

In this study, we aimed to present our institution’s experience with concurrent IHR during RARP.

## 2. Materials and Methods

### 2.1. Population

We retrospectively reviewed 452 consecutive patients who underwent RARP for PCa between January 2018 to December 2020 in our institution. Inclusion criteria were adult patients with histological diagnosis of adenocarcinoma of the prostate who had submitted to RARP as their chosen primary treatment option, and a pre- or intra-operative diagnosis of IH. Patients with a previous history of IH repair or bowel in the hernia sac were excluded from the analysis. All patients with symptomatic IH were previously evaluated by physical examination and abdominal ultrasound. A single expert robotic urologist performed concomitant RARP and IHR in the same sitting.

### 2.2. Data Collection

The following data were collected: age, body mass index (BMI), smoking habits, diabetes, prostate-specific antigen (PSA), prostate volume, the American Society of Anesthesiologists (ASA) score, intra-operative data (i.e., operative time, estimated blood loss, length of hospital stay, catheter and drainage dwelling time), and any early postoperative complications (i.e., within 30 days). Early complications were ranked according to the Clavien–Dindo (CD) classification [16]. During the postoperative period, patients were managed for pain using an infusion pump consisting of 6 vials containing 30 mg/mL of Ketorolac, and two vials containing 20 mg/mL of morphine, for 48 h with a rate of 2 mL/h. Patients were assessed at 3-, 12-, and 24-month post-operative follow-up intervals for IH recurrence, mesh infection, seroma formation, and groin pain with physical examination and abdominal ultrasound. The presence of symptoms, including fever, hyperemia, swelling, and groin pain, was considered suspicious for mesh infection. The study followed the principles of the Declaration of Helsinki and its amendments. This study was conducted retrospectively, with prospective data collection from daily clinical practice, and all the procedures were performed as part of routine care.

### 2.3. Surgical Technique

All IHR procedures were performed after the traditional transperitoneal anterior RARP approach with the Montsouris technique and modified posterior musculofascial reconstruction by Rocco et al. [17]. Anastomosis integrity was evaluated by injecting 160 mL of sterile sodium chloride into the bladder. In patients with an IH, the peritoneal leaves were opened, the hernia content was reduced, and the sac was dissected. The patented transverse fascia was pulled, and monofilament polypropylene mesh (Herniamesh^®^ S.r.l., Chivasso, Torino, Italy) was used to repair the defect. Thereafter, the mesh was fixed using non-absorbable sutures (Ethibond 0, Ethicon, Somerville, NJ, USA), and the peritoneum was applied and closed over the mesh to avoid bowel adhesions.

### 2.4. Statistical Analysis

Categorical variables were expressed as frequency and percentage (%), and continuous variables were assessed as median and interquartile range (IQR).

## 3. Results

After records screening, a total of 73 patients undergoing simultaneous RARP and IHR were included. Patients’ characteristics are summarized in Table 1. The median age was 67 years (IQR 56–77), and the median prostate volume was 38 mL (IQR 25.0–75.2). The median ASA score was 2 (IQR 1–3). 38 patients (52.0%) had IH on the right side, and 31 (42.5%) on the left side, while in the remaining 4 cases (5.5%) the defect was bilateral. In 58 (79.5%) cases, IH was direct, and in 15 (20%) indirect. Inguinal pain/discomfort was present in 33 (45.2%) patients. Fifty-eight (79.4%) patients were diagnosed as having an inguinal mass before surgery. IH was diagnosed at surgery in 11 (15.1%) patients.

The peri-operative outcomes are reported in Table 2. IHR was performed successfully in all cases. Bilateral standard pelvic lymph node dissection was performed in 21 patients (28.8%). The median total surgical time and the IHR time were 190.0 (IQR 140.0–230.0) and 32.5 (IQR 14.0–40.0) minutes, respectively. The median estimated blood loss was 100 mL (IQR 10–170). The median catheterization and drainage time were 8 (IQR 6–12) and 2 days (IQR 1–3), respectively; the median length of stay was 3 days (IQR 2–4). Only 5 (6.8%) minor complications occurred: 2 patients (2.7%) had a fever that required paracetamol infusion, and 3 patients (4.1%) had a post-operative decrease in hemoglobin over 2 g/dL which required no transfusion (all CD grade 1 complications). No complications CD 2 or higher were recorded.

The median postoperative follow-up was 24 months (IQR 17–32), and there were no cases of mesh infection, seroma formation, or lymphocele. IH recurrence was reported in one case (1.4%).

## 4. Discussion

IH and PCa are both commonly diagnosed in aging men, and there is a relatively high rate (13–33%) of concomitant incidence [18,19]. In light of this, clinicians need to perform a thorough clinical examination before performing surgery for PCa, to ensure that any inguinal hernia is detected and appropriately managed. Moreover, IHR during RP is often recommended, including when incidentally identified during RP, since it may become symptomatic following surgery. Based on our experience, we have observed that 73 out of 452 (16%) patients who underwent RARP had IH, which is in line with data present in the literature [10].

IH may require surgical intervention due to symptoms or complications, such as bowel obstruction and strangulation, although the risk is generally low [20]. Elective IHR is commonly performed for chronic pain, and this has traditionally been done through an open or laparoscopic approach. Delayed IHR following RP can be challenging due to scarring tissue in the preperitoneal space. The operative time for subsequent IHR ranges in the literature from 45–111 min [21], which is longer than ours. This confirms that IHR during RARP may be considered less challenging than after RP, at least for procedural time.

Yet, concomitant IHR during RP surgery has shown several other advantages, including single anesthesia, and avoiding potential technical difficulties in repairing the hernia through laparoscopic or robotic surgery due to scarring [22].

Whenever prosthetic materials are used in surgical procedures, complications associated with the use of these materials may occur, including meshes for IHR. Furthermore, the risks associated may vary depending on the type of material used, the size and location of the implant, and the patient’s overall health status. Their use is associated with potential risks, including infection and bowel adhesions. Endoscopic approaches have shown a low incidence of complications, such as infections and hematoma formation, particularly when compared to open mesh repairs [23]. Our results confirm that IHR during RARP was not associated with bleeding or infectious complications, confirming that robotic-assisted laparoscopic HR is a safe procedure to be performed during RARP.

The risk of infection arises from the possibility of the mesh coming into contact with urine or intraperitoneal organisms during surgery [24]; however, it is estimated to be low, at 0.17%, after laparoscopic IHR [25]. Moreover, the usage of absorbable meshes may reduce the risk of infection, although the efficacy of these meshes is still under investigation [26].

Bowel adhesion is another potential complication associated with the use of prosthetic meshes, although quite uncommon [27]. Adhesion-resistant meshes and their fixation are strategies recommended and used to prevent adhesions, as well as the closing of the anterior peritoneum over the mesh to reduce the risk of bowel adhesion. In our cohort, we had no bowel adhesion, likely because we closed the peritoneum over the mesh.

Other potential postoperative complications after the same-sitting RARP and IH may occur. The development of seroma is a common complication after the laparoscopic approach, with incidence rates up to 20% [28]. However, studies on the robot-assisted technique have reported no occurrence of seroma formation: proper techniques, such as ensuring good hemostasis, effective drainage of surgical site fluids, and running absorbable suture use can prevent the development of seroma [29]. Secondly, lymphocele formation can occur due to prolonged lymphatic drainage after pelvic lymph node dissection. However, the incidence of lymphoceles requiring drainage during minimally invasive RP and concurrent IH repair was reported to be less than 5% [30]. Endoclips, rather than cautery application during lymph node dissection, may reduce its incidence. Our study found no cases of seroma or lymphocele formation during the follow-up, and only one patient (1.4%) experienced a recurrence of an IH. Finally, although it is unusual, patients undergoing simultaneous surgeries may experience inguinal, scrotal, or testicular pain or paresthesia [31]; in our series, no cases experienced these complications.

Several studies have investigated the feasibility of performing concurrent IHR during RARP. Finley et al. found this procedure safe and feasible, with an average operative time of only 8 min. One recurrence and no complications were reported in their series of 40 patients [32]. Another study by Xia et al. supported the safety of this combined surgery in patients with PCa and IH; in their case series of 357 patients, using standardized definitions of 30-day adverse events, there was no increase in postoperative complications rate between RARP alone and RARP with concurrent IHR [33]. Similarly, Lee et al. reported no recurrence at the site of repair with a laparoscopic approach; the low morbidity of elective IHR also supports the idea that it does not increase the risk of postoperative adverse events [34]. Of note, a large, randomized study comparing the open to the laparoscopic mesh IHR found that the latter had a higher recurrence rate and a greater risk of serious complications [35]. In our cohort, only five patients experienced peri-operative minor complications, yet all were not linked to IHR, confirming that robot-assisted IHR is a safe procedure.

In terms of pain medication requirements after surgery, previous studies have shown controversial results, with some reporting no significant difference and others an increased usage of analgesics, especially among patients who underwent transperitoneal rather than extraperitoneal procedures [36]. Based on the data of our study, only 2 patients required paracetamol medication.

Our study is not devoid of limitations. First, it is a retrospective study and biases linked to its nature are predictable. Second, a relatively small sample size was involved. Finally, in this analysis there was no control group with staged RARP followed by IHR.

## 5. Conclusions

Our study confirms that concomitant IHR and RARP surgeries are feasible and safe. Although concomitant IHR increases the surgical operating time, in our case series no high-grade peri-operative complications related to IHR were observed, and no long-term adverse events occurred, with only one case of IH recurrence. Therefore, simultaneous surgery can eliminate the need for a second procedure, with subsequently reduced healthcare costs.

## Figures and Tables

**Table 1:** Patients baseline characteristics. BMI = body mass index; PSA = prostatic specific agent; IHR = inguinal hernia repair; RARP = robot-assisted radical prostatectomy; ASA = American Society of Anesthesiologists. IQR = interquartile range.

Variable	Concomitant IHR with RARP (73 pts)
Age, year, median (IQR)	67 (56–77)
Smoking habits, n (%)	18 (24.7)
Diabetes/HTA, n (%)	8 (10.9)
BMI, kg/m^2^, median (IQR)	27.4 (25.0–31.0)
ASA Score, n (%)	2 (1–3)
Pre-operative PSA value, ng/mL	7.8 (2.6–23.0)
Inguinal pain/discomfort at presentation, n (%)	33 (45.2)
Inguinal hernia presentation, n (%)	
Discovered at surgery	11 (15.1)
Inguinal mass	58 (79.4)
Unknow/missing data	4 (5.5)
Prostate volume, cc, median (IQR)	38.0 (25.0–75.2)
IH side, n (%)	
Left	31 (42.5)
Right	38 (52.0)
Bilateral	4 (5.5)
IH location, n (%)	
Direct	58 (79.5)
Indirect	15 (20.5)

**Table 2:** Intra- and peri-operative outcomes. IHR = inguinal hernia repair; RARP = robot-assisted radical prostatectomy; CD = Clavien–Dindo; ISUP = International Society of Urological Pathology. IQR = interquartile range.

Variable	Concomitant IHR with RARP (73 pts)
Operative time, minutes, median (IQR)	190.0 (140.0–230.0)
IHR time, minutes, median (IQR)	32.5 (14.0–40.0)
Estimated blood loss, mL, median (IQR)	100.0 (10.0–170.0)
Length of hospital stay, days, median (IQR)	3 (2–4)
Duration of drainage, days, median (IQR)	2 (1–3)
Duration of catheterization, days, median (IQR)	8 (6–12)
Complications, n (%)	
CD 1	5 (6.8)
CD ≥ 2	0 (0)

## Data Availability

The data presented in this study are available on request from the corresponding author. The data are not publicly available due to privacy issues.
